# Antibiotic Transport in Resistant Bacteria: Synchrotron UV Fluorescence Microscopy to Determine Antibiotic Accumulation with Single Cell Resolution

**DOI:** 10.1371/journal.pone.0038624

**Published:** 2012-06-12

**Authors:** Slávka Kaščáková, Laure Maigre, Jacqueline Chevalier, Matthieu Réfrégiers, Jean-Marie Pagès

**Affiliations:** 1 DISCO beamline, Synchrotron Soleil, Saint-Aubin, France; 2 UMR-MD1, Aix-Marseille Université, Transporteurs Membranaires, Chimiorésistance et Drug-Design, IRBA, Facultés de Médecine et de Pharmacie, Marseille, France; University of Birmingham, United Kingdom

## Abstract

A molecular definition of the mechanism conferring bacterial multidrug resistance is clinically crucial and today methods for quantitative determination of the uptake of antimicrobial agents with single cell resolution are missing. Using the naturally occurring fluorescence of antibacterial agents after deep ultraviolet (DUV) excitation, we developed a method to non-invasively monitor the quinolones uptake in single bacteria. Our approach is based on a DUV fluorescence microscope coupled to a synchrotron beamline providing tuneable excitation from 200 to 600 nm. A full spectrum was acquired at each pixel of the image, to study the DUV excited fluorescence emitted from quinolones within single bacteria. Measuring spectra allowed us to separate the antibiotic fluorescence from the autofluorescence contribution. By performing spectroscopic analysis, the quantification of the antibiotic signal was possible. To our knowledge, this is the first time that the intracellular accumulation of a clinical antibitiotic could be determined and discussed in relation with the level of drug susceptibility for a multiresistant strain. This method is especially important to follow the behavior of quinolone molecules at individual cell level, to quantify the intracellular concentration of the antibiotic and develop new strategies to combat the dissemination of MDR-bacteria. In addition, this original approach also indicates the heterogeneity of bacterial population when the same strain is under environmental stress like antibiotic attack.

## Introduction

Since the use of antimicrobial agents to combat bacterial infections, the emergence of bacteria that are resistant to antibiotics was observed [1]–[8]. As a result, a large number of infectious diseases, which had been reported to be controlled or totally eradicated, are again in an upswing. Bacteria involved in re-emerging infectious diseases increasingly withstand the action of antibiotic clinical therapies by the dissemination of multi-drug resistance (MDR) mechanisms [1]–[6]. The worldwide spreading of MDR pathogens limits the treatment options and has become a major cause of the therapeutic failures and mortalities in hospital wards during recent decades. Obviously, MDR has became a major health concern worldwide (for specific websites see: World Health Organization: http://www.who.int/drugresistance/en/; Center for Disease Control and Prevention: http://www.cdc.gov/drugresistance/index.html; European Food Safety Authority: http://www.efsa.europa.eu/en/topics/topic/amr.htm; European Center for Disease Prevention and Control: http://www.ecdc.europa.eu/en/healthtopics/antimicrobial resistance/Pages/index.aspx.

Extensive research has identified three major bacterial strategies involved in the MDR development: (i) the target protection barrier (alteration/modification of the target site); (ii) the enzymatic barrier (degradation of the antibiotic molecule) and (iii) the membrane barrier (acting to limit the required intracellular dose of antibiotic) [9]. The drug resistance caused by the membrane barrier, the first defence line in bacterial cells, is now recognized to be a synergy between a reduced drug influx (due to lowering of membrane permeability, *e.g.* by modification of porin activity [9]) and an active efflux of the drug (via efflux pumps that expel the antibiotic out of the cell before it can reach its target site). As consequence, the concentration of antibiotics within bacteria decreases under the threshold required for its activity [9]–[14]. This reduction can contribute to the development of additional mechanisms of resistance including mutation of antibiotic targets (*e.g.* in gyrase) or production of enzymes that cleave antibiotics (*e.g.* ß-lactamases), and also reinforces the efficacy of these acquired mechanisms [9], [12], [13]. In the face of this continuously emerging threat, the development of efficient strategies to circumvent the bacterial MDR responses depends upon understanding the molecular basis of the mechanism controlling the intracellular concentration of antibacterial agents.

A key point is the accurate determination of the antibiotic concentration inside the targeted bacterium. Several experimental approaches have been developed to reach this goal. Antibiotics determination in biological samples has been performed by radiometry [15]–[17], bioassays [18], [19] and by UV absorbance and fluorescence emission on samples separated by high-pressure liquid chromatography (HPLC) [20], [21]. While bioassay techniques are too laborious and generally inappropriate for kinetic studies, the HPLC requires several manipulations such as deprotonation and ion-pair extraction for sample preparations. Moreover, UV absorption methods require higher concentrations of antibiotics, since the UV detection is limited to concentrations in the microgram/milliliter range. Radiometric assays are sensitive and accurate, however they require drug radiolabeling, which could impair biological activity of the molecule and requires an internal standard. To overcome the limitations of existing methods, the fluorimetric method has been proposed by Chapman and Georgopapadakou [22]. The method is based on the natural fluorescence of clinically used antibiotics, *e.g.*, fluoroquinolones such as ciprofloxacin or levofloxacin. A solubilization of bacteria by lysate buffer is required prior to fluorescence detection of antibiotics. Showing high sensitivity, rapidity and minimal sample manipulation, the fluorimetric method is an attractive alternative to the more invasive bioassay or less sensitive UV absorption assay. However, the remaining problem associated with any of these assays is that they detect antimicrobial agent in overall bacterial masses, giving no specific information on antibiotic concentration within any single cell. Knowledge about the intracellular concentration and antibiotic location inside the bacterium is essential to define the complete molecular mechanism and the possible ways for tackling antibiotic resistance.

Here we report the quantitative single-cell fluorescence microscopic study of *Enterobacter aerogenes* resistant strains using a new non-invasive method preserving the antibiotic structure which have allowed us to monitor the antibiotic uptake depending on the efflux pump activity. Considering the natural fluorescence of clinically used quinolone, we use synchrotron radiation DUV imaging and the synchrotron radiation DUV microspectroscopy as new methods to investigate the drug accumulation inside individual bacteria. Two fluorescence microscopes were used: for DUV imaging we used a DUV compatible full-field microscope, whereas the DUV microspectroscopy was achieved by using the microscope, which allows collecting the fluorescence spectra. Fleroxacin (Fle) was chosen as target quinolone to test the concept *in vitro*. Since the fluorimetric method of Fle uptake on digested bacterial masses has been previously validated [22], [23], we used this method as our internal standard. The intrabacterial concentration has been studied in two bacterial strains of *Enterobacter aerogenes*: a resistant isolate EA289 that overproduced the broad spectrum AcrAB-TolC efflux pump, and the *tolC* deficient derivative strain EA298 [24]. The activity of efflux pump on antibiotic uptake has been assessed using co-incubation with glucose (Glu) as well as carbonyl cyanide m-chlorophenyl hydrazone (CCCP). CCCP is a powerful uncoupler of the proton motive force (PMF) that collapses the membrane energy, consequently, used at low concentrations, it inhibits the drug transport through the inner membrane. Belonging to the group of efflux pump blockers/modulators, it is used for a long time to study the antibiotic expel by Gram-negative efflux pumps (for recent reviews see [25], [26]). These results have important implications for the understanding of intracellular accumulation of quinolones in single multidrug resistant clinical bacteria and to develop original ways to combat resistance mechanisms associated with membrane permeability.

## Results

To investigate the mechanism that controls the intracellular concentration of antibiotics in single resistant isolate and to define the ways to increase normal concentration, we have selected a tri-fluoroquinolone, flerofloxacin (fleroxacin, Fle) and an *E. aerogenes* resistant strain from a MDR isolate which overexpresses the broad spectrum AcrAB-TolC efflux pump and its efflux derivative (*tol*C^-^). Glucose (Glu) and carbonyl cyanide m-chlorophenyl hydrazone (CCCP) are used to respectively energize and collapse the active efflux transport. The incubation time was selected from the previous experiments carried out on this resistant isolate with radiolabelled norfloxacin indicating that the steady-state level of fluoroquinolone accumulation was observed at about 10 min incubation [27].

### Antibiotics Concentration Measured by Fluorimetric Assay on Bacterial Lysates

**Figure 1:**
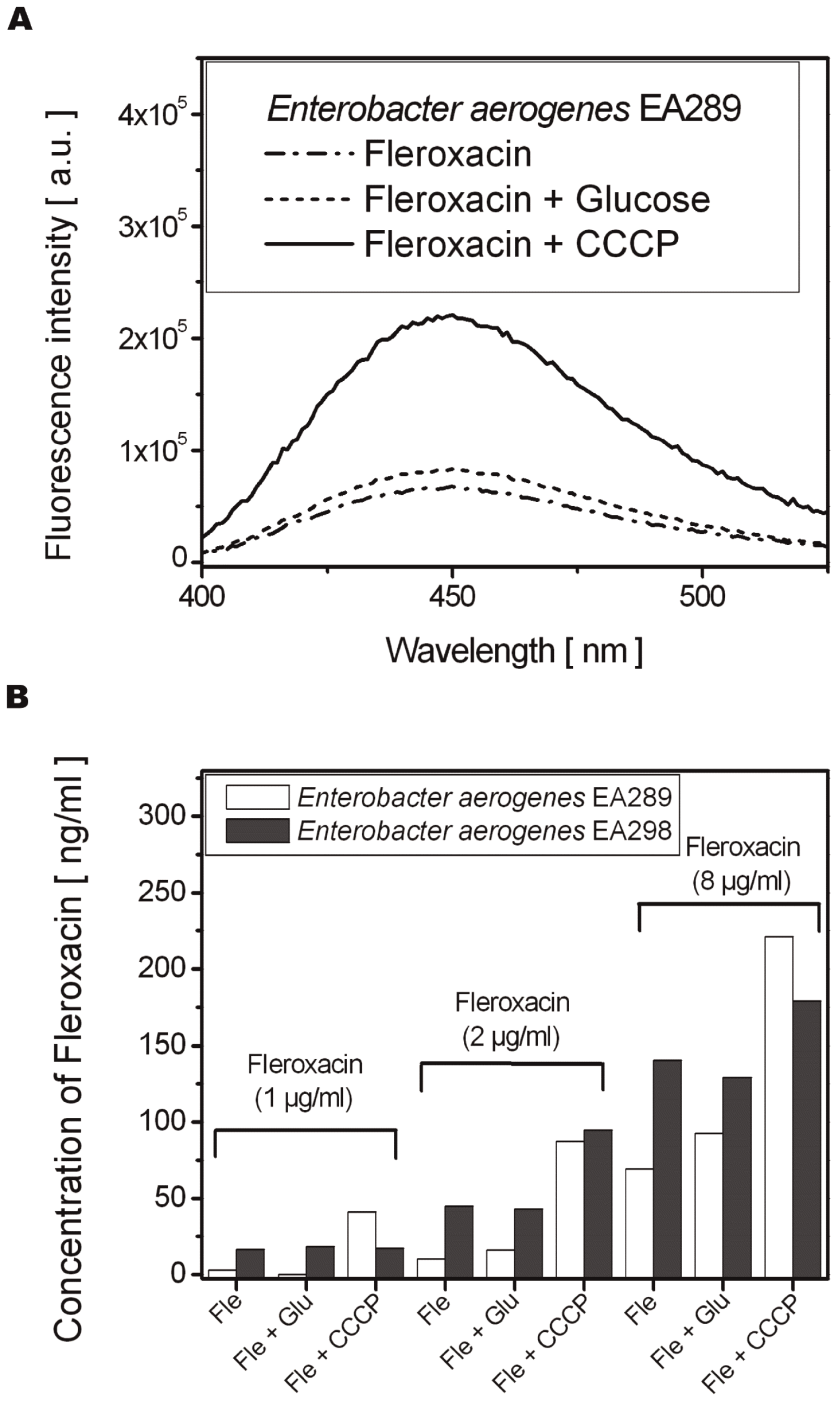
Fleroxacin uptake by *Enterobacter aerogenes* population. A . Fluorescence emission spectra of Fle (λ_exc_ = 283 nm) detected from Glycin-HCl-induced lysis of *Enterobacter aerogenes* strain EA289. EA289 were incubated with Fle (2 µg/ml) for 30 min in follow conditions: (− ·− ·) Fle; (−−−) Fle + Glu (0.4%); (–) Fle + CCCP (10 µM). **B**. Comparison of Fle concentration uptake determined from lysated bacteria. *Enterobacter aerogenes* strains EA289 and EA298 were incubated with Fle at different concentrations (1, 2 or 8 µg/ml) alone or with Glu (0.4%) or CCCP (10 µM).

The dependence of Fle fluorescence as a function of bacteria co-incubation (+CCCP, +Glu or in the absence of additives) is presented in **Fig. 1A**. Incubation of the bacterial suspension with CCCP increased the Fle uptake by bacterial cells, *i.e.* a higher fluorescence intensity of Fle was detected, when compared with the incubation carried out with antibiotic alone. In contrast, no significant difference in Fle fluorescence was observed during co-incubation with or without Glu. Note, the maximum of fluorescence spectra emission from **Fig. 1A** corresponds to the emission of Fle fluorescence previously identified in lysate buffer (Gly-HCl, pH = 3) (**Fig. S1**).

For each condition, the Fle fluorescence intensity was connected with its concentration based on calibration curve. The concentration of Fle uptake by bacteria is presented in **Fig. 1B**. Starting with Fle concentration of 1µg/ml, which represents the detection limit of this fluorimetric assay (Fig. 1B), the intracellular drug concentration increases, with higher Fle concentrations. This trend was observed for resistant as well as sensitive strains of *E. aerogenes*. Furthermore, in the absence of Glu or CCCP, for each concentration of Fle incubation, the intracellular Fle concentration was higher in EA298 (sensitive) than in EA289 (resistant). This fits well with Fle-MIC measured for the two strains (**Table 1**). The effect of CCCP, added as uncoupler of membrane energy during antibiotic incubation, boosted intracellular concentration of Fle (**Fig. 1B**). This increase was more important in efflux-producer strain EA289. The similar results to those for Fle have been observed also for another clinically relevant quinolone, ciprofloxacin (Cip). Cip accumulation was more pronounced in EA298 than in EA289. Cip concentration was similarly increased in the presence of CCCP for both strains (more pronounced for EA289 than for EA298) and the Glu effect varied between Cip concentrations and bacteria strains (**Fig. S2**).

**Table 1:** Major characteristics of bacterial strains and MIC values for fluoroquinolones.

Bacterial strains	Characteristics	MIC (µg.ml^−1^)	
*Enterobacter aerogenes*		Fle	Cip
EA289	Kan^s^ derivate of EA27 (MDR clinical isolate)	128	32
EA298	EAEP289 *tol*C^-^ :Kan^r^	32	4

Fle, fleroxacin; Cip, ciprofloxacin.

### Single Cell DUV Fluorescence Microspectroscopy and Imaging

**Figure 2:**
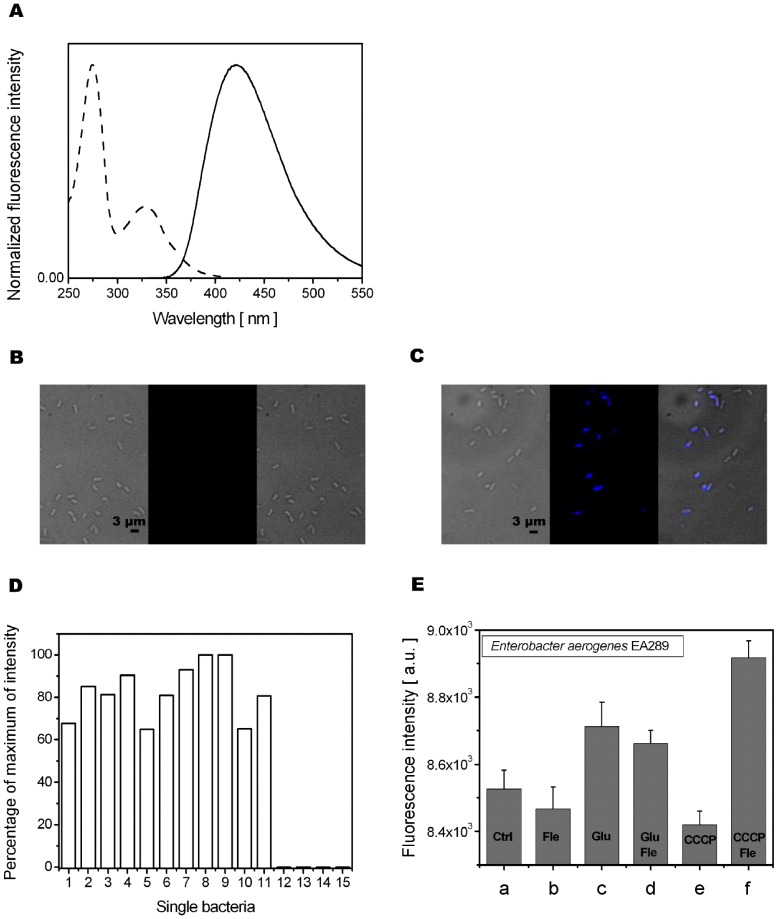
Fleroxacin uptake by individual *Enterobacter aerogenes*. A . Fluorescence spectra of Fle (750 ng/ml) in PBS pH = 7: (−−−) excitation spectrum at λ_emis_ = 420 nm; (–) emission spectrum at λ_exc_ = 313 nm. **B**. Transmission (left), fluorescence (middle) and merge (right) images of Fle (64 µg/ml)-treated EA289 bacteria. Scale bar corresponds to 3 µm. **C**. Transmission (left), fluorescence (middle) and merge (right) images of Fle (64 µg/ml)- and CCCP- (25 µM) treated EA289 bacteria. Scale bar corresponds to 3 µm. **D**. Percentage of maximum fluorescence intensity of Fle within single bacteria from **fig. 2C. E**. Fluorescence intensity detected from Fle channel (λ_exc_ = 290 nm; DM 300 nm; BP filter 420≤ λ_emis_ ≤480 nm) from individual EA289 bacteria as a function of treatment conditions: (a) EA289 with no additions; (b) EA289 incubated with Fle (64 µg/ml); (c) EA289 incubated with Glu; (d) EA289 co-incubated with Glu and Fle (64 µg/ml); (e) EA289 incubated with CCCP (25 µM); (f) EA289 co-incubated with CCCP (25 µM) and Fle (64 µg/ml).

In order to determine experimental conditions to detect Fle fluorescence in bacteria, spectral characteristics of Fle were analysed. The fluorescence emission and excitation spectra of Fle in PBS, pH = 7 are presented in **Fig. 2A**. In this buffer, Fle exhibits a maximum of fluorescence emission at a wavelength, λ_emis_, of 421 nm and a maximum of excitation at a wavelength, λ_exc_, of 275 nm. To assess the optimal excitation wavelength for fluorescence detection of Fle, we had to carefully consider the contribution of natural fluorophores present in bacteria, notably tyrosine, tryptophan and NADH molecules. Bacterial natural fluorophores could exhibit fluorescence intensities orders of magnitude greater than those from the weakly fluorescence antibiotics. Excitation at λ_exc_ = 290 nm revealed to be the best for the Fle detection in living bacteria. The fluorescence emission was detected in the λ_emis_ = 420−480 nm wavelength range. However, as presented on **Fig. 2A**, this covers only half of the Fle fluorescence. This energy band pass was selected maximise the contribution of Fle relative to the autofluorescence of tryptophan, the main contributor to the overall autofluorescence signal.

Because microscopy technique deals with single bacteria the concentration of Fle was adjusted to 64 µg/ml. **Figs. 2B–2C** present transmission and fluorescence images of EA289 strain obtained in Fle-treated bacteria (at 64 µg. ml^−1^ concentration) without additives (**Fig. 2B**) and in the presence of CCCP (**Fig. 2C**), after subtraction of the autofluorescence contribution. No fluorescence was detected from Fle-treated bacteria (**Fig. 2B**). This is in contrast to Fle-treated bacteria co-incubated with CCCP (**Fig. 2C**) where a high signal was detected. We attribute this to a marked increase of intracellular Fle fluorescence. In addition, from the merge image of the transmission and fluorescence images in **Fig. 2C**, it can be noted that some individual bacteria were not fluorescent even in the presence of proton uncoupler. **Figure 2D** shows the quantitative distribution of Fle fluorescence from single bacteria presented on **Figure 2C**. Signal variations from single cells (**Fig. 2D**) indicates that inside a bacterial population, issue from a same controlled strain inoculum, different level of resistant phenotypes may co-exist controlling the antibiotic accumulation, as recently reported by Lee and co-workers under external stress [28] (for a review see [29]).

In **Fig. 2E**, the fluorescence intensities of the 420–480 nm wavelength range from EA289 bacteria strain incubated with and without Fle in presence of Glu and CCCP are presented. While Glu induced increase in bacterial autofluorescence when compared with un-treated bacteria, the signal co-incubated with CCCP was lower. Considering Glu as energizer, whereas CCCP as proton uncoupler [4], [30], it is not surprising that the optical properties of bacteria changed as a result of metabolic activity modification. The sensitivity of DUV microscopy for changes in the signal can be seen also for Fle. Results from **Fig. 2E** suggest that the measurement of Fle accumulation with DUV microscopy must be corrected by the bacterial autofluorescence contribution and the variation between the different induced autofluorescences due to incubation conditions. Only Fle-CCCP-treated bacteria (in **Fig. 2E**, noted the difference F-E) demonstrated significantly higher intensity in 420–480 nm wavelength range when compared to control conditions (in **Fig. 2E**, noted the difference B–A or D–C).

### DUV Microspectrofluorimetry

**Figure 3:**
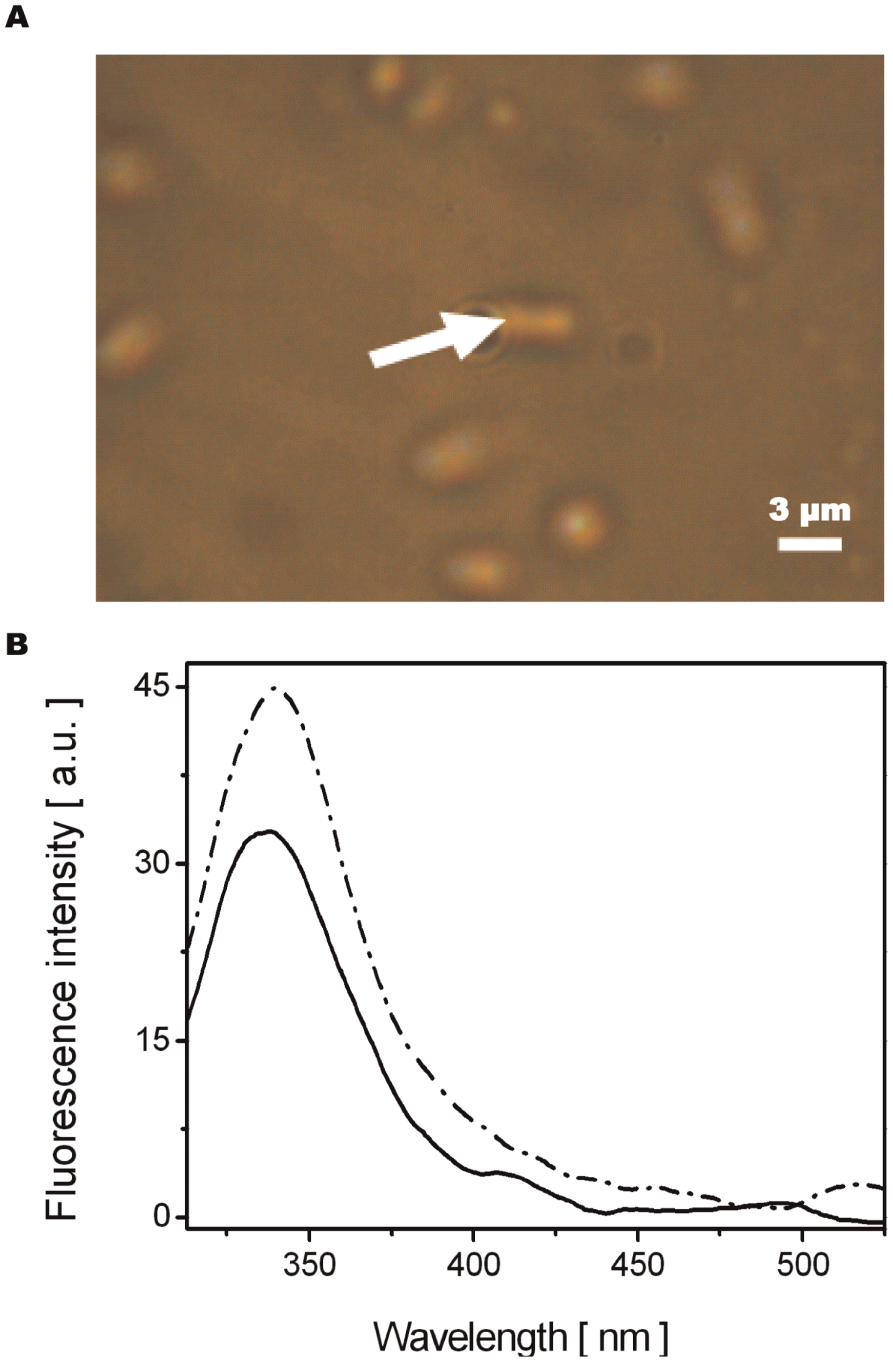
Individual bacteria microspectro-fluorescence measurement. A . Transmission image of *Enterobacter aerogenes* EA289 bacteria. White arrow indicates on bacterium from which one of the fluorescence spectra of **fig. 3B** was taken. Scale bar corresponds to 3 µm. **B**. ****Fluorescence emission spectra (recorded by UV-VIS microspectrofluorimetry at λ_exc_ = 290 nm) from two individual Fle-untreated bacteria EA289. Fluorescence emission spectrum (− ·− ·) corresponds to bacterium marked on **fig.** **3A**; (–) spectrum corresponds to bacterium not in the field of view.

Spectra recorded from single Fle-untreated bacteria (indicated in **Fig. 3A**) with λ_exc_ = 290 nm are presented in **Fig. 3B**. The peak around λ_emis_ = 340 nm corresponds to the tryptophan fluorescence, an aromatic amino-acid, component of proteins [31]–[33]. The comparison of those spectra shows differences in inter-bacterial auto-fluorescence intensity (**Fig. 3B**). To correct for those differences, spectra from individual bacteria from different locations were averaged and then normalized on the tryptophan peak.

**Figure 4:**
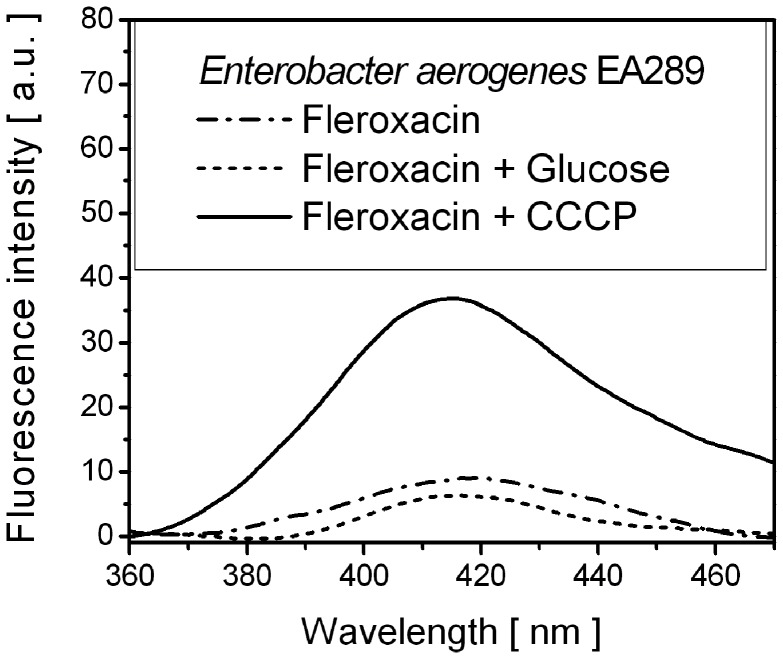
Fluorescence intensity of Fleroxacin from *Enterobacter aerogenes* bacteria. Mean fluorescence emission spectra (λ_exc_ = 290 nm) of Fle from *Enterobacter aerogenes* EA289 bacteria were measured by UV-VIS microspectrofluorimetry. Fle (64 µg/ml)-treated bacteria were co-incubated with follow additives: (–) CCCP (25 µM); (−−−) Glu (0.4%) and (− ·− ·) in the additives absence (EA289 incubated only with Fle).

Control and Fle spectra were thereafter compared. Spectra are presented as Supplementary information (**Figs. S3A–C**). Fle increased the fluorescence recorded between 400 and 450 nm. Subtraction of control spectra revealed Fle fluorescence emission spectra (**Fig. 4**). The peak position of fluorescence emission of Fle fits well with Fle fluorescence in PBS buffer at pH = 7 (**Fig. 2A**) meaning that spectral behaviour of the drug were not significantly modified in bacteria.

**Figure 5:**
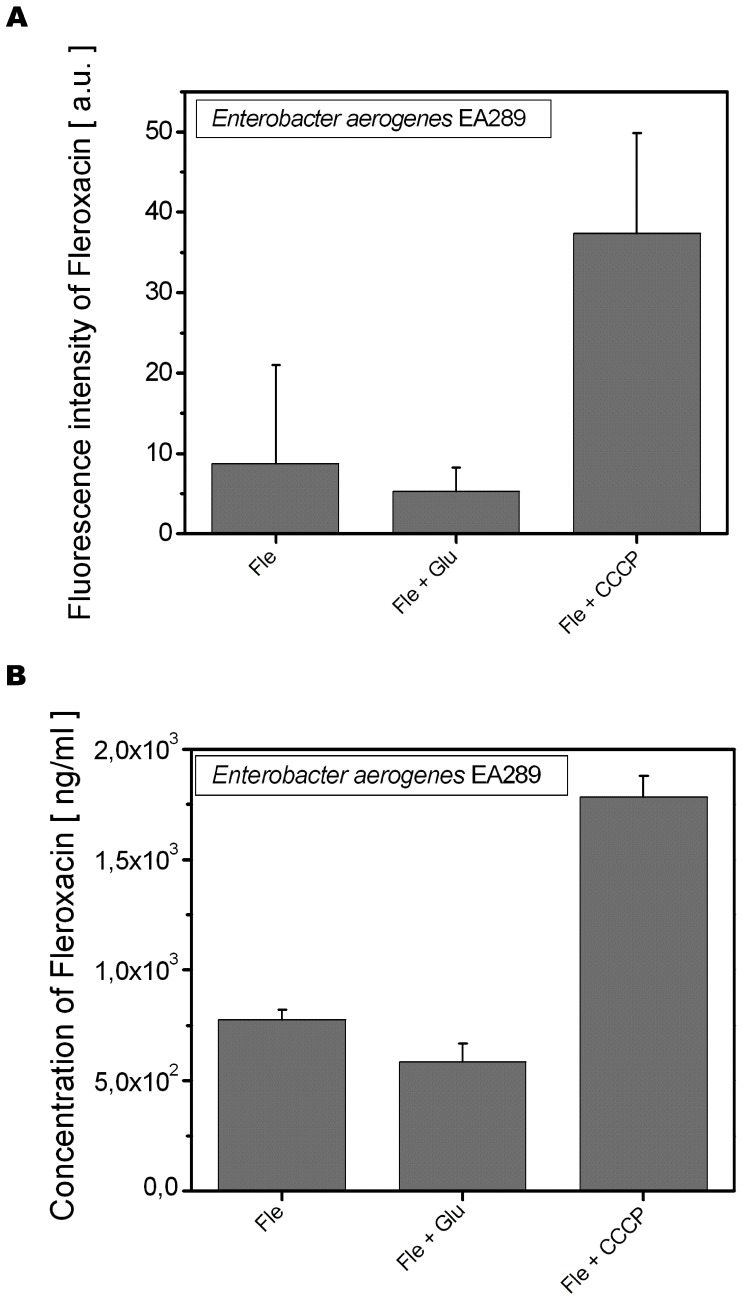
Comparison of fluorescence intensity and concentration of Fleroxacin in individual bacteria and lysated bacteria. A . ****Fluorescence intensity of Fle measured by UV-VIS microspectrofluorimetry from individual EA289 bacteria. EA289 were incubated with Fle (64 µg/ml) only or with Glu (0.4%) or CCCP (25 µM). **B**. ****Comparison of Fle concentration uptake determined from lysated bacteria. *Enterobacter aerogenes* EA289 were incubated with Fle (64 µg/ml) alone or with Glu (0.4%) or CCCP (25 µM).

Analyses of the Fle fluorescence obtained from different locations of bacteria showed a decrease within Glu-treated bacterial samples, while the co-incubation with CCCP demonstrated significantly higher fluorescence than with Fle alone (**Fig. 5A**). Similar variations in Fle uptake demonstrated by extraction method are presented on **Fig. 5B**.

By using radiolabelled norfloxacin to investigate the intracellular drug concentration in a different *E. aerogenes* resistant clinical isolate, Chevalier *et al* had reported a rough increase of fluoroquinolone when the incubation was carried out in the presence of a similar concentration of CCCP, the ratio CCCP-treated/non-treated bacteria being 2.3 [34]. It must be noted that during this previous determination, no internal control was used to standardize the assays and no test was carried out with Glu addition.

## Discussion

With the continuing emergence of MDR bacteria, methods have been developed to follow the uptake of antibiotics in bacteria, but none is presently able to quantify the quinolones uptake in single bacteria. Indeed, the knowledge about the intracellular concentration of antibiotics and their accumulation inside the bacterial cell is a key point to molecularly dissect the resistance mechanism and develop possible ways for tackling MDR bacteria. In addition, it is now recognized that small internal concentrations of drugs, under the threshold necessary to kills bacteria, are able to induce the accumulation of internal metabolites (signals) that triggers the bacterial response and activate genetic cascade of resistance mechanisms [9], [23], [28], [30].

For these reasons, it is now urgently required to develop the concept of quinolones detection in single bacteria using the DUV fluorescence microspectroscopy and imaging. This new method is validated by detection of quinolone molecule Fle for which an extraction measurement was derived from a previously established protocol [22], [23]. In addition, to assess a direct measurement of efflux pump activity and correlation with Fle bacterial susceptibility, the differences in Fle uptake within an *E. aerogenes* isolate that overproduces efflux pump, EA289, and an efflux-derivative strain, EA298, were investigated.

A higher antibiotic uptake was determined in the *tolC* derivative EA298, when compared with the parental strain EA289 that over-expresses AcrAB-TolC efflux pump. To distinguish whether this is due to the activity of efflux pump in the latter strain, the measurement was carried out with CCCP, an uncoupler known to collapse the membrane energy and block the energy-dependent efflux pump [35]–[37]. A substantial increase in the Fle uptake by EA289 in the presence of CCCP clearly illustrates the contribution of efflux pump on the drug concentration. Furthermore, this accumulation was higher than when EA298 were incubated in absence of the efflux pump inhibitor. Our observations with another clinically relevant quinolone, ciprofloxacin (Cip) (**Fig. S2**) are in concordance; Cip accumulation was more pronounced in EA298 than in EA289. Cip concentration was similarly increased in the presence of CCCP for both strains (more pronounced for EA289 than for EA298) and the Glu effect varied between Cip concentrations and bacteria strains.

The fluorescence detection by DUV imaging was focused on EA289 bacteria for which the gain of the intracellular concentration of Fle in the presence of CCCP was more prominent. In addition, the Fle concentration of 64 µg/ml was selected to allow comparison between the steady-state spectrofluorimeter and the fluorescence microscopes, respecting their minimum fluorescence detection limits (*i.e.*, 64 µg/ml in DUV analysis compared to 1 µg/ml in fluorimetric detection within a bacterial lysate). Indeed, while 10^9^ cells/ml contributed to fluorescence which is detected from bacteria lysate, only one cell contributed to fluorescence detected by imaging system.

To assess the Fle uptake in individual bacteria we used two DUV fluorescence microscopes: the first microscope collects fluorescence spectra of microvolumes, and the second, full-field microscope gives the global fluorescence intensity in a defined spectral range (420–480 nm in this study). We would like to emphasize that the eligibility of an antibiotic for synchrotron-radiation UV fluorescence microspectroscopic studies depends upon production of post-excitation fluorescence between 200 and 400 nm, associated with a minimal interference due to bacterial autofluorescence emission spectra. The second condition is difficult to achieve since antibiotics exhibit small Stoke’s shift and because several natural occurring fluorochromes exhibit absorption in the UV and DUV region [33], [37]. To minimize the contribution from the autofluorescence signal, we had to carefully consider the absorption and fluorescence properties of natural fluorophores present in bacteria, notably tyrosine, tryptophan and NADH. In our study, the selected excitation wavelength λ_exc_ = 290 nm was the best for obtaining a high Fle/autofluorescence ratio. This wavelength corresponds to a minimum of absorption for bacterial autofluorophores [33] and justifies our observation of highest Fle/autofluorescence contrast. In addition, in order to reduce the contribution of autofluorescence, the fluorescence of Fle was analyzed in the 420–480 nm spectral range. For the first time, detection of Fle inside single bacteria was possible. It is important to note that the Fle detection is impaired by autofluorescence contribution that changes with the incubation conditions. This has important consequences for the general interpretation of fluorescence measurements. The autofluorescence contribution plus its correlation with physiological processes in bacteria are making the quantitative approach of Fle fluorescence difficult. A method correcting for the influence of autofluorescence effects, would be a step forward.

We used synchrotron-radiation DUV microspectroscopy as a method to accurately quantify the antibiotic fluorescence in single bacteria. Measuring the spectra from single bacteria allowed monitoring of antibiotic fluorescence together with autofluorescence contribution. By doing spectroscopic analysis, the autofluorescence was successfully subtracted and consequently, the quantification of the signal from different conditions of co-incubations was possible.

The comparison of the antibiotic fluorescence measured on single bacteria with the levels obtained from bacteria lysates yielded strong evidence that the concentration uptake of antibiotics (Fle and Cip) was heavily increased by CCCP addition. This proves that synchrotron-radiation UV fluorescence microspectroscopy can be used as a method to evidence the antibiotic intra-bacterial accumulation and thus directly monitor the efflux pump activity and the antibiotic susceptibility correlation.

In addition, we clearly demonstrated that the fluorescence drug signal from individual bacteria varied, within in a uniformly treated population. Considering that measurements are done on single bacteria, our technique can be further developed and applied to probing a bacterial population, and generate statistical information about individual bacterial uptake since isogenic microbial populations contain substantial cell-to-cell differences in physiological parameters such as growth rate, resistance to stress and regulatory circuit output (for a review see [29]). Regarding this point, it is important to mention that Long *et al* [38] recently reported that the quantification by single-cell fluorescence microscopy of bacterial signalling responses to AI-1 and AI-2 autoinducers involved in quorum sensing regulation, indicates a coherent response across the population but some cell-to-cell variations. This is especially important taking into account the bacterial adaptability to external stresses and antibiotic attacks and reflects the recent observation about the bacterial charity [28]. In addition, this result correlates with the large and rapid change involving the expression of transporters in bacterial membrane faced with external antibacterial agents (including antibiotics, biocides, etc). This efficient alteration of transporters expression rapidly modifies the membrane permeability and the associated-drug uptake [39], [40]. This original approach indicates the heterogeneity of bacterial population regarding the antibiotic accumulation and may provide appropriate clues to understand the role of ciprofloxacin in the bacterial adaptation and persister formation as recently reported by Dörr *et al* [41].

To our knowledge, this is the first time that an antibiotic under its natural clinical form (*e.g.* without artificial labelling) has been monitored within single bacterial cells. This is a matter of importance taking into account recent publication reporting that low antibiotic concentration are able to select and maintain resistance mechanisms in bacterial population and several mathematical models analysing antibiotic combinations to treat resistant bacteria [42], [43]. This places synchrotron-radiation UV fluorescence microspectroscopy in an ideal position to quantify the uptake of non-modified clinical antimicrobial agents in bacteria, and open a new field of research in antibacterial chemotherapy.

This first study represents an important milestone for understanding and combating the drug resistance mechanism in MDR bacteria. Important questions remain to be addressed in subsequent studies. It will be extremely valuable to determine the location of antibiotics during their uptake inside bacterial cells, in membrane, in periplasmic space or in cytoplasmic space, and how the inhibition of drug transporters changes this location. In addition, it will be important to determine the concentration and location of antibiotic molecules targeting the bacterial membrane.

## Materials and Methods

### Bacterial Strains and Growth Conditions

Strains used in this study are listed in Table 1. Experiments were carried out on two *E. aerogenes* strains previously described [24]: EA289, a clinical multi-drug resistant strain that overexpresses AcrAB-tolC efflux pumps, and EA298, its *tol*C^-^ derivative. Strains were routinely grown at 37°C on Luria-Bertani (LB) agar or in LB broth, supplemented with kanamycine (50 µg.ml^−1^) for EA298.

### Antibiotics Accumulation

Bacteria grown in its exponential-phase (corresponding to 0.6 optical density units at 600 nm) were concentrated 10 fold. Briefly, the bacterial suspension was centrifuged at 6 000 g for 15 min at 20°C and pellets were resuspended in 1/10 of the volume in a sodium phosphate buffer (50 mM) at pH 7 supplemented with MgCl_2_ (NaPi-MgCl_2_ buffer) to obtain a density of 10^10^ CFU.ml^−1^. Bacteria suspension (1.6 ml) was incubated 30 min at 37°C (final volume 2 ml) with different concentration of antibiotics, Fle or Cip (1, 2, 4, 8 or 64 µg.ml^−1^) in the absence or in the presence of CCCP (10 µM or 25 µM) or Glu (0.4%) respectively. Bacterial suspensions incubated without antibiotics, with CCCP or with Glu were used as controls. Suspensions (800 µl or 400 µl) were then loaded on 1 M sucrose cushion (1100 µl or 550 µl respectively) and centrifuged at 13 000 rpm for 5 min at 4°C to eliminate extracellular-adsorbed fluoroquinolones and collect washed bacteria.

To follow the Fle uptake by EA289 and EA298 strains, we used the routine fluorimetric method previously described by Chapman *et al*. [22]. Briefly, pellets corresponding to 800 µl of suspensions were solubilized with 500 µl of 0.1 M Glycin-HCl pH3 buffer at least 2 h at room temperature. After a centrifugation for 10 min at 13 000 rpm, 400 µl of lysates were diluted in 600 µl of 0.1 M Glycin-HCl pH3 buffer and analysed by spectrofluorimetry.

To detect the antibiotics fluorescence from single bacteria, pellets corresponding to 400 µl of suspensions were resuspended in 200 µl of NaPi-MgCl_2_ buffer and analysed by DUV microspectrofluorimetry or DUV fluorescence imaging.

### Spectrofluorimetry Analysis

The fluorescence spectra of bacteria lysates were recorded at 20°C using a FluroMax-4 (HORIBA Jobin Yvon INC, Chilly Mazarin, France) spectrofluorimeter. The quartz cuvette of 1 cm pathlength was used for measurement. Fluorescence emission spectra of lysate were recorded at an excitation wavelength of 283 nm. This wavelength was previously determined to be suitable for Fle detection (*e.g.* in 0.1 M buffer of Glycin-HCl of pH = 3, Fle exhibits its maximum of excitation at 283 nm and emission at 451 nm (**Fig. S1**)). To quantify the Fle fluorescence intensity in bacteria lysate, spectra were normalized using the tryptophan peak at 356 nm (**Fig. S4**) and subtraction of spectra representing control samples (no drug, Glu or CCCP only) was proceed. Fle concentrations in bacteria lysate were calculated according to a calibration curve generated by mixing a known concentration of Fle with Glycin-HCl of pH = 3.

### DUV Microspectrofluorimetry

Fluorescence spectra from individual bacteria were recorded on a deep ultraviolet (DUV) microspectrofluorimeter at Synchrotron SOLEIL [31]. Briefly, the 290 nm excitation of a bending magnet from DISCO beamline [44] was focalised on bacteria deposited with their medium (0.5 µl of resuspended pellets) on quartz coverslips. The objective used was Zeiss ultrafar immersed objective with 100× magnification. Emission spectra were recorded with the acquisition time of 90 s and have been measured on 5 different localisations, *i.e.* 5 different bacteria. Individual spectra recorded in the same condition from bacteria on different localisations were smoothed and averaged by Microcal Origin, version 8.0 program (Microcal Software, Inc., Northampton; MA). To compare the region of antibiotic fluorescence, spectra were brought on the same baseline level and normalized on the peak of tryptophan at 340 nm (**Fig. S3**).

### DUV Fluorescence Imaging

Bacteria strains were observed in brightfield and excitated in DUV with a Zeiss Axioobserver Z−1. The selected objective was a 100× Zeiss ultrafluar objective needing glycerine immersion. The Fle fluorescence was recorded by excitation at 290 nm, using the dichroic mirror of 300 nm (OMEGA Optical, Inc., USA) and emission bandpass filter for 420–480 nm wavelengths (OMEGA Optical, Inc., USA). To detect fluorescence images, we used CCD camera from Hamamatsu C9100-13 (HAMAMATSU PHOTONICS France SARL, France). The integration time of camera was 2 min for applied excitation and filter combination used to visualize the Fle fluorescence. In addition, to increase the signal to noise ratio, images were recorded with binning of the pixel (2×2) in Micro-Manager program [45], used to manipulate the CCD camera.

The image analyses were performed with Image J (Rasband, W.S., ImageJ, U. S. National Institutes of Health, Bethesda, Maryland, USA, http://imagej.nih.gov/ij/, 1997–2011). Illumination being in-homogeneous, it was corrected before background subtraction. First, threshold was automatically adjusted using a triangle algorithm; thereafter, bacteria were analysed as the remaining particles. The mean intensity coming from each bacterium was automatically calculated considering its pixel area. Finally, all bacteria signal taken from one image were averaged. For each condition, two different localisations with minimum 30 bacteria per plane were recorded and averaged. Resulting fluorescences were analysed using Microcal Origin 8 (Microcal Software Inc, Northampton, MA).

In order to present images of bacteria with fluorescence signal from Fle, the contribution of autofluorescence to the instinct fluorescence of bacteria was measured from Fle-untreated bacteria with follow subtraction from Fle-treated bacteria (*e.g.* bacteria incubated with Fle in CCCP absence and/or presence). The same contrast was chosen for all images for better visual comparison.

## Supporting Information

Figure S1 **Fluorescence spectra of Fle (50 ng/ml) in Glycin–HCl pH = 3.** (−−−) excitation spectrum at λemis = 451 nm; (–) emission spectrum at λexc = 283 nm.
(TIFF)

Figure S2 **Comparison of Ciprofloxacin concentration uptake determined from lysated bacteria.** Enterobacter aerogenes strains EA289 and EA298 were incubated with Cip (1, 2 or 4 µg/ml) alone or with Glu (0.4%) or CCCP (10 µM).
(TIFF)

Figure S3 **Fluorescence spectra of Fleroxacin from individual bacteria incubated with different additives.** A.Mean fluorescence emission spectra (λ_exc_ = 290 nm) of control (–) and Fle-treated (–) Enterobacter aerogenes EA289 bacteria measured individually by UV-VIS microspectrofluorimetry. B.Mean fluorescence emission spectra (λ_exc_ = 290 nm) of control (–) and Fle-Glu-treated (–) Enterobacter aerogenes EA289 measured individually by UV-VIS microspectrofluorimetry. C.Mean fluorescence emission spectra (λ_exc_ = 290 nm) of control (–) and Fle-CCCP-treated (–) Enterobacter aerogenes EA289 measured individually by UV-VIS microspectrofluorimetry.
(TIFF)

Figure S4 **Fluorescence emission spectra (λ_exc_ = 283 nm) of** ***Enterobacter aerogenes*** **strain EA289 bacteria lysate.** Bacteria were incubated with or without Fle (2 µg/ml) in follow conditions: (−−−, red) no additions; (−··−··, red) with CCCP (10 µM); (−−−, blue) with Fle and Glu (0.4%); (–, blue) with Fle; (−··−··, black) with Fle and CCCP (10 µM). Bacteria incubated with Glu demonstrates the identical spectra features as bacteria with no additions, e.g. (−−−, red).
(TIFF)
